# Kinematic model calibration of a collaborative redundant robot using a closed kinematic chain

**DOI:** 10.1038/s41598-023-45156-6

**Published:** 2023-10-18

**Authors:** Tadej Petrič, Leon Žlajpah

**Affiliations:** Department of Automatics, Biocybernetics and Robotics, Jožef Stefan Institute, Jamova cesta 39, 1000 Ljubljana, Slovenia

**Keywords:** Electrical and electronic engineering, Mechanical engineering

## Abstract

In this paper, we propose a novel approach for the kinematic calibration of collaborative redundat robots, focusing on improving their precision using a cost-effective and efficient method. We exploit the redundancy of the closed-loop kinematic chain by utilizing a spherical joint, enabling precise definition of the robot end-effector position while maintaining free joint motion in the null space. Leveraging the availability of joint torque sensors in most collaborative robots, we employ a kinesthetic approach to obtain constrained joint motion for calibration. An optimization approach is utilized to determine the optimal kinematic parameters based on measured joint positions and a constrained end-effector position defined by the spherical joint. The effectiveness of the proposed method is demonstrated and validated on the Franka Emika Panda robot, a 7-DoF robot. Results indicate a significant enhancement in absolute accuracy, with comparable performance to more expensive sensor systems such as optical measurement systems. Our approach offers a practical and cost-effective solution for improving the precision of collaborative robots.

## Introduction

Robots have revolutionized the manufacturing industry, offering numerous advantages over traditional methods, including greater efficiency, precision and productivity. They are particularly useful in mass production, where repetitive tasks can be easily automated by recording and repeating specific movements. However, programming robots for low-volume production can be difficult and time-consuming because the production is typically smaller and more diverse. To overcome this obstacle, offline programming strategies have been developed in which the robot is programmed without using the actual machine. This approach can significantly reduce programming time and eliminate the need for trial-and-error programming. However, offline programming requires precise robot kinematics to ensure that the programmed motions are executed accurately on the actual robot. Robot manufacturers typically provide nominal kinematic models of their robots using Denavit–Hartenberg (DH) parameters. However, these models may not be completely accurate due to various factors such as manufacturing and assembly errors. In practice, the lack of precise knowledge of robot kinematics and inaccuracies in the nominal models can limit the practical use of robots in low-volume applications.

Many online and offline methods have been developed to minimize kinematic error and enable more practical use of robots^1,2^. All these methods have in common that they need a reference measurement system, on which the calibration quality depends, as can be seen in Table 1, where the pros and cons of the most typical measurement systems for calibration are compared.

**Table 1:** Comparison of different measurement systems for kinematic identification (adapted from^3^). Note that the highlighted line represents our new proposed approach.

Devices	Rep.	MC	Port.	Cost
Theodolite	5 mm	Static	High	Medium
Ultrasonic	1 mm	Dynamic	High	Low
Machine vision	1 mm	Dynamic	High	Medium
Laser tracker	10 μm	Dynamic	High	High
IR MoCap	0.1 mm	Dynamic	Medium	Medium
Spherical joint	0.1 mm	Dynamic	High	Low

*Rep.* Repitability, *MC* measurement characteristic, *Port.* portability.

Recent studies on the kinematic calibration of industrial robots have explored various approaches and techniques. One common approach is iterative optimization based on least-square theory, aiming to enhance calibration accuracy^4^. Another method combines geometric principles with dual vector algebra, providing a refined geometric and dual vector algebra approach for kinematic parameter refinement^5^. Additionally, a combination of geometric and parametric methods has been proposed to improve the calibration process^6^. Researchers have also investigated alternative methods for kinematic parameter identification, such as utilizing monocular cameras to estimate parameters by analyzing the robot’s motion and corresponding images^3^. Indexed measurement platforms have been utilized to capture specific measurements from the robot’s movements, aiding in the identification of kinematic parameters^7^. Furthermore, error models have been developed specifically for flexible robots, considering deformations and uncertainties in the robot structure^8^. Investigations have even been conducted on the identification of kinematic parameters for collaborative robots on mobile platforms using motion capture systems^9,10^.

In terms of novel approaches, researchers have introduced a vision-based calibration framework that utilizes visual information to refine kinematic parameters in industrial robotic manipulators^11^. They proposed a method that combines geometric and parametric approaches for the identification of kinematic parameters in industrial robots^6^. Moreover, a precise calibration solution has been presented by utilizing a single telescoping ballbar for absolute robot calibration^12^. An alternative calibration approach was explored through the investigation of non-kinematic calibration of a six-axis serial robot using planar constraints^13^. Furthermore, a metrological device specifically designed for accurate robot identification and calibration measurements has been developed^14^. Despite the diverse range of approaches, it is worth noting that many of these methods either rely on expensive measuring devices or are not suitable for redundant robots.

To address the limitations mentioned above, we propose a kinematic calibration approach specifically tailored for collaborative redundant robots. Our methodology capitalizes on the utilization of a closed-loop kinematic chain facilitated by a spherical joint. While the realm of kinematic calibration has been extensively explored, the majority of prevailing techniques mandate the use of external measurement tools like laser trackers or specialized mechanical apparatuses. In contrast, our approach circumvents the necessity for such equipment by harnessing the inherent capabilities of a closed-loop kinematic chain coupled with a spherical joint. This pivotal departure significantly mitigates the financial burden associated with the acquisition of elaborate instruments, while concurrently ensuring that the accuracy of the calibration is predominantly contingent upon the precision of the spherical joint itself. Notably, this not only enhances the affordability of the overall calibration process but also bolsters the system’s portability, rendering it more adaptable for various operational settings.

In our proposed approach, the kinematic calibration of collaborative redundant robots is achieved by leveraging a closed-loop kinematic chain. This configuration constrains the motion of the robot’s end-effector. To gather the necessary data for calibration, we employ two methods: manual motion generation through kinesthetic guidance or prescribed motion using a null-space controller. Notably, this approach enables data capture at the sampling frequency of the robot, facilitating accurate calibration.

By collecting these measurements, we can apply the optimization method outlined in^10^ to precisely determine the Denavit–Hartenberg (DH) parameters. Unlike many existing approaches that focus on correcting the final end-effector pose, our method is specifically designed for redundant robots, such as typical collaborative robots with 7 degrees of freedom (DOF). For such robots, introducing corrections to the end-effector pose becomes impractical. Therefore, our approach offers a suitable solution for effectively calibrating these robots and optimizing their kinematic parameters.

The contribution of this work, going beyond our study in^10^, may be summarized as follows: (i) employing a spherical joint and thereby capitalizing on the redundancy within the closed-loop kinematic chain to calibrate the robot; (ii) an analysis of the number of different spherical joint fixtures and required joint range in the null-space needed for identification; (iii) experimental evaluation for DH parameter estimation in simulation and real robot.

The paper is organized as follows. In "Errormodeling,measurementproceduresandparameteroptimization: Section, we give an overview of the kinematic modeling of a robot, measurement procedures and algorithms for the identification of DH parameters. In "Analysisin simulation" Section we provide results from simulation analysis and in "Results ofrobotcalibration" Section, we provide results and compare different calibration strategies on a real robot. Conclusions can be found in "Conclusion" Section.

## Error modeling, measurement procedures and parameter optimization

**Figure 1:**
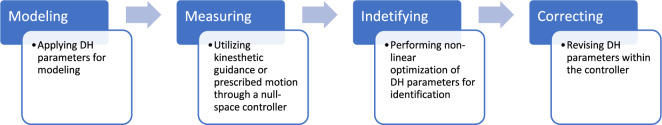
Flow diagram illustrating the sequential steps of the proposed calibration process for collaborative robots using closed-loop kinematic chains and spherical joints.

Typically, the process of calibrating robots using models involves the following steps as shown in Fig. 1: modeling, measuring, identifying, and correcting. These steps will be elaborated upon in the following sections.

### Modeling

The Denavit–Hartenberg (DH) convention is commonly used to analyze a robotic manipulator’s motion. This convention uses a set of four parameters, denoted $P_{DHi}=(a_i,\alpha _i,d_i,\theta _i)$, to define the homogeneous transformation matrix ${^{i-1}\textbf{T}_{i}}$. This matrix describes the transformation between two adjacent reference frames attached to the links $i-1$ and *i* connected by the joint *i*. Assuming that joint *i* is a revolute joint, which means it rotates about a single axis, the variable $q_i$ represents its position. The matrix ${^{i-1}\textbf{T}_{i}}(q_i)$ is then calculated based on the values of the DH parameters and the joint position $q_i$. This matrix describes the transformation of the coordinate system of the link *i* relative to the link $(i-1)$ due to the motion of joint $q_i$. For revolute joints, it is given by

1 $$\begin{aligned} {^{i-1}\textbf{T}_{i}}(q_i,P_{DHi})={{\,\textrm{R}\,}}_{{\varvec{z}}_{i-1}}(\theta _{i}+q_{i})\cdot {{\,\textrm{L}\,}}_{{\varvec{z}}_{i-1}}(d_{i})\cdot {{\,\textrm{L}\,}}_{{\varvec{x}}_{i}}(r_{i})\cdot {{\,\textrm{R}\,}}_{{\varvec{x}}_{i}}(\alpha _{i}) \end{aligned}$$

where ${{\,\textrm{L}\,}}_{\varvec{a}}(\cdot )$ and ${{\,\textrm{R}\,}}_{\varvec{a}}(\cdot )$ represent the translation and rotation along vector ${\varvec{a}}$, respectively. Note that the DH parameters also have physical meanings: $a_i$ represents the distance between the $z_{i-1}$ and $z_i$ axes, measured along the $x_i$ axis; $\alpha _i$ represents the angle between the $z_{i-1}$ and $z_i$ axes, measured about the $x_i$ axis; $d_i$ represents the distance between the $x_{i-1}$ and $x_i$ axes, measured along the $z_{i-1}$ axis; $\theta _i$ represents the angle between the $x_{i-1}$ and $x_i$ axes, measured about the $z_{i-1}$ axis.

By using the DH convention and calculating the corresponding transformation matrices for each joint of a robot, we can obtain the complete kinematic model of the robot with

2 $$\begin{aligned} {^{0}\textbf{T}_{E}}({{\varvec{q}}},P_{DH})=\left( \prod _{i=1}^{n}{^{i-1}\textbf{T}_{i}}(q_i,P_{DHi})\right) {^{n}\textbf{T}_{E}}= \left[ \begin{array}{cc} {\textbf{R}}&{} {\varvec{p}}\\ \textbf{0} &{} 1 \\ \end{array}\right] . \end{aligned}$$

here *n* refers to the number of joints in a robotic manipulator. The matrix ${^{n}\textbf{T}_{E}}$ represents the fixed transformation between the robot flange and the end-effector, which is the part of the robot interacting with the environment. The variables ${\varvec{p}}$ and ${\textbf{R}}$ denote the position and orientation of the end-effector, respectively.

Errors in kinematic parameters can lead to discrepancies between the actual and the expected position and orientation of the robot’s end-effector. Let us assume that the robot manufacturer provides nominal DH parameters denoted as $P_{DHi}=(a_i,\alpha _i,d_i,\theta _i)$, which are used to model the robot’s kinematics. However, due to various sources of error such as manufacturing tolerances or wear and tear, the actual DH parameters may differ from the nominal ones, denoted as $\widehat{P_{DH}}_i=(\hat{a}_i,\hat{\alpha }_i,\hat{d}_i,\hat{\theta }_i)$. As a result, the position and orientation of the end-effector will deviate from the expected values due to these kinematic errors. To quantify the magnitude of these errors, we can calculate the difference between the actual and nominal end-effector poses with

3 $$\begin{aligned} {{\varvec{e}}}_p={\varvec{p}}({{\varvec{q}}},\widehat{P_{DH}})-{\varvec{p}}({{\varvec{q}}},P_{DH}), \end{aligned}$$

4 $$\begin{aligned} {{\varvec{e}}}_R=\log ({\textbf{R}}({{\varvec{q}}},\widehat{P_{DH}}){\textbf{R}}({{\varvec{q}}},P_{DH})^T) . \end{aligned}$$

In summary, errors in kinematic parameters can lead to significant discrepancies between the actual and nominal end-effector poses, and it is important to quantify and minimize these errors to ensure accurate and reliable robotic operation. Note that in the hereafter, only the ${\varvec{p}}$ position of the robot’s end-effector is considered for calibration using the proposed method.

**Figure 2:**
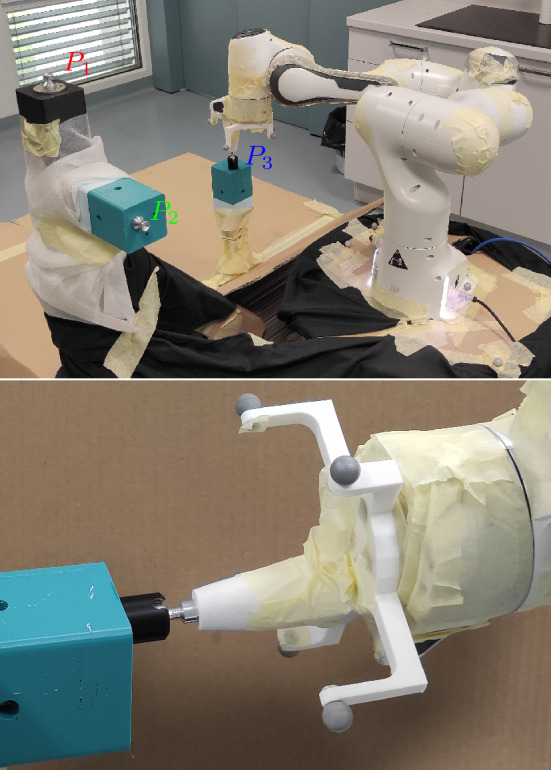
Experimental setup with three spherical joint fixture points in top picture and zoom-in with robot attached to P2 in bottom picture.

### Measurements

In order to improve the accuracy of the end-effector’s position, it is necessary to determine the actual kinematic parameters of a robot. This requires collecting data that relates the robot’s inputs to its outputs. A common method for calibration is to move the robot to several known configurations where joint positions and end-effector positions are measured. The selection of joint configurations depends on the method used for parameter calculation. For example, a geometric approach based on known joint axes and linear algebra requires each joint to be moved independently to determine the joint axes and relative frames for each joint, which are necessary to calculate DH parameters^9,10,15,16^. However, this approach is only suitable if the robot’s end-effector can move freely in space.

An alternative is to measure enough joint configurations that define a well-conditioned set for parameter estimation^17^. There are various proposals for selecting joint configurations^18,19^. As proposed in^10^, one possibility is using random configurations throughout the robot’s workspace or in some subspace when there are motion restrictions.

Considering that the aforementioned methodologies frequently entail the utilization of high-cost measurement equipment or involve a slow data acquisition process, in response to these constraints and in pursuit of ameliorating the current paradigm, our proposed calibration method uses a spherical joint to constrain the motion of the robot’s end-effector. While the spherical joint only constrains the position, the end-effector orientation is not constrained. By defining the 3-dimensional end-effector position as the primary task, the robot gains three additional functional redundant degrees-of-freedom (DOF). This redundancy allows the robot to be moved to different configurations for calibration. It is important to select spherical joint locations where the resulting self-motion of the robot allows a large range of joint motions so that the obtained measurement set is well-conditioned. If the robot is intrinsically redundant, i.e. it has more than 6 DOFs, and the null-space is much larger, the situation is much better. If still necessary, data can be collected for more than one location of the spherical joint. Note that for the proposed method, the robot’s end-effector position is preserved during measurements.

The process for determining this position of selected points is as follows: In scenarios where optimization was performed with only one point, we derived the end-effector position by taking the average of all the measured points obtained through computations using nominal DH parameters. This approach allowed us to obtain a representative position estimate. However, in cases where multiple points were incorporated, for example for Pall where all three designated points (P1, P2, and P3) were utilized for optimization, we introduced an additional layer of precision. Here, we factored in the precise knowledge of the relative distances between these points. By incorporating this information, we could further refine the end-effector position determination for calibration, ensuring a more accurate representation of the robot’s end-effector location.

Measurement accuracy is a critical factor in calibration. In the proposed spherical joint constraint method, the position of the robot’s end-effector is not directly measured because the joint is supposed to be fixed. However, during measurements, it may be observed that the end-effector is still moving. This could be due to mechanical properties of the spherical joint such as backlash, or deflections of the fixture of the spherical joint due to forces between the robot and the joint. These effects can be considered as measurement noise.

### Identification

If the robot end-effector is constrained, only an optimization-based approach can be used for the identification of Denavit–Hartenberg (DH) parameters^20,21^. This is because the DH parameters describe the kinematic properties of the robot’s links and joints, and their values directly affect the position and orientation of the end-effector. One of the possible methods we have used in this paper is Particle Swarm Optimization (PSO). Detailed information regarding the convexity and convergence of the problem for this method can be found in^22,23^. However, it is important to note that while we have chosen PSO for our research, other optimization methods can also be applied for DH parameter identification. The objective function to be minimized is typically defined as the error between the measured end-effector positions and the calculated positions using the robot’s forward kinematics. The optimization problem can then be expressed as

5 $$\begin{aligned} \{P_{DH}\} = \arg \min _{P_{DH}} \sum _{k=1}^m \left( \Vert {\varvec{p}}({{\varvec{q}}}_k,P_{DH})-{\varvec{p}}_{M,k}\Vert \right) , \end{aligned}$$

where *m* is the number of measured samples, ${\varvec{p}}_{M,k}$ and ${{\varvec{q}}}_k$ are the measured end-effector positions and joint positions for sample *k*, respectively. $P_{DH}$ represents the DH parameters of the robot. The optimization process requires multiple samples of the robot configuration, where the number of samples is denoted by *m*. In the case of the spherical joint constraint, the position of ${\varvec{p}}_{M,k}$ is a constant for all *k* in a given position. Note that in our case we used different fixed positions, as shown in Fig. 2.

### Correction

The last stage of the calibration process is correction. If the robot controller permits the direct modification of the model’s kinematic parameters, the correction step is straightforward. However, in our particular scenario, we had to employ novel direct and inverse kinematics techniques within the controller, which were based on the updated kinematic model.

## Analysis in simulation

To evaluate the calibration process, we used a simulation environment. Our simulation environment includes the versatile physics engine MuJoCo^24^, which allowed us to create a model of the Franka Emika Panda robot. This involved representing the robot as a sequence of interconnected links and joints, utilizing the available DH parameters. We also developed a control system using Matlab to control the robot’s movements and collect data about its state. Using Matlab allowed us to program the robot’s movements with ease and track its progress, making the entire process more streamlined and efficient.

In simulation, we have generated robot self-motion for data collection automatically. For that, the robot kinematic controller was adjusted to exploit the redundant DOFs. Redundant DOFs result from intrinsic redundancy and functional redundancy. Our robot has 7 DOFs, i.e., it has one intrinsic redundant DOF. As we are controlling only the end-effector’s position, the system has three additional redundant DOFs. So, four robot DOFs can be used for self-motion. To prevent constraint violation, we allowed only self-motion of the robot, i.e., the commanded joint velocities were selected as

6 $$\begin{aligned} {\dot{{{\varvec{q}}}}}_d = {\textbf{J}}\textbf{K}_p ({\varvec{p}}_B-{\varvec{p}}) + {\textbf{N}}{\dot{{{\varvec{q}}}}}_r = {\textbf{N}}{\dot{{{\varvec{q}}}}}_r , \end{aligned}$$

where ${\varvec{p}}_B$ is the fixed position of the spherical joint, ${\textbf{N}}$ is the null-space of the position part of the Jacobian ${\textbf{J}}={\textbf{J}}_p$ and ${\dot{{{\varvec{q}}}}}_r$ were random joint velocities. Note that the end-effector is constrained to a fixed position. Therefore, the position error in (6) always equals 0. Note also that ${{\varvec{q}}}_r$ does not affect the end-effector position. With such motion control we generated motion sequences where the robot was moving around the fixed end-effector position. To obtain a well-conditioned data set we had to exploit the available null-space motion as much as possible. Therefore, in our validation, the random velocities ${\dot{{{\varvec{q}}}}}_r$ were changed every two seconds. These increased the possibility of making the most of the null-space.

**Figure 3:**
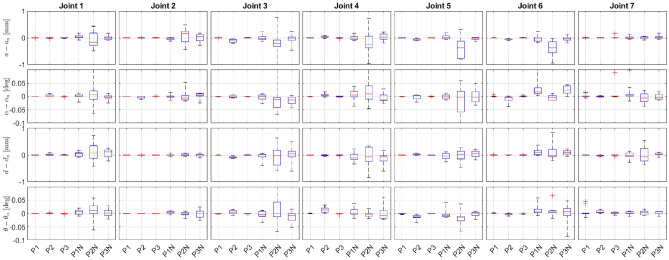
Statistics of DH parameter estimation—Deviation of $\widehat{P_{DH}}$ parameters from their nominal values $P_{DH}$ for all three points: without a measurement noise (P1, P2, and P3) and with added measurement noise (P1N, P2N, and P3N).

**Figure 4:**
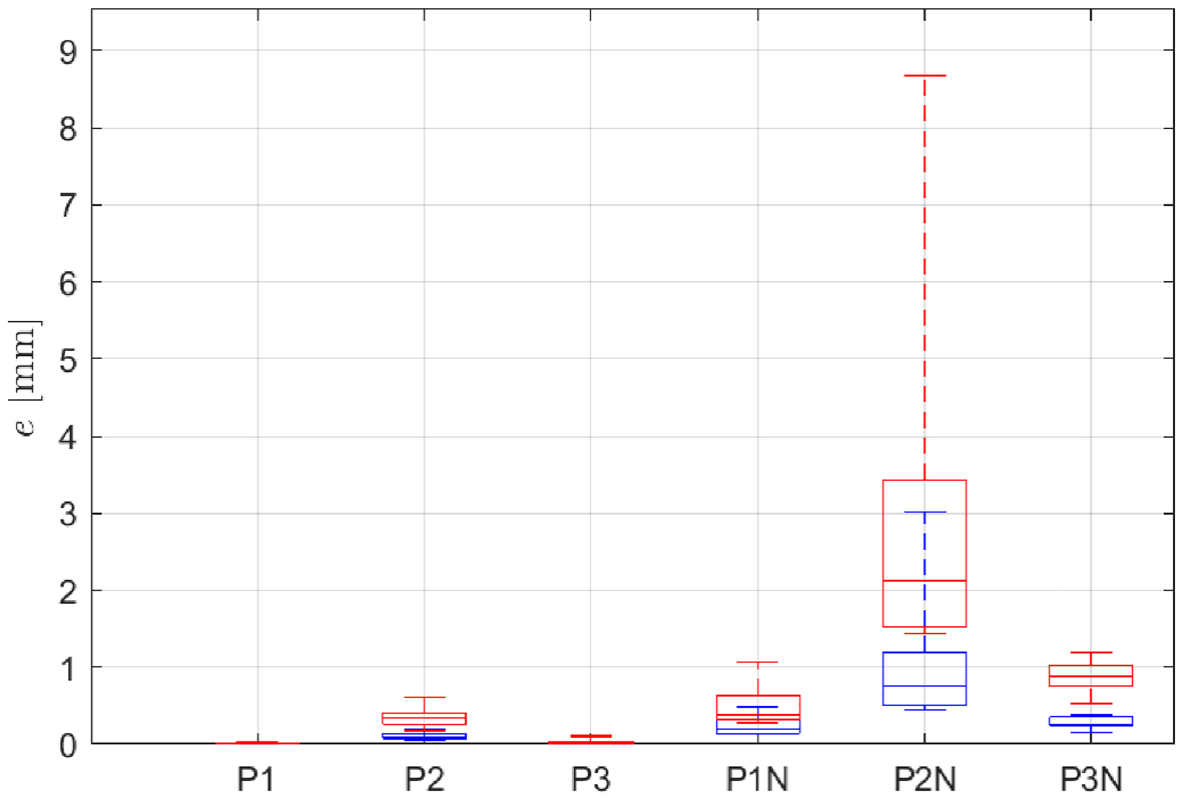
Statistics of the end-effector position error: average position error statistics (blue) and maximal position error statistics (red).

To test the validity of the proposed calibration method, we generated several different motion sets in the simulator for each of the three points (P1, P2, and P3) shown in Fig. 2. Using the generated data we prepared 10 motion sets, and for each set we have selected randomly 500 configurations. The robot parameters were set to their nominal values, and the optimization process was initiated using incorrect DH parameters. This approach was adopted to confirm the effectiveness of the calibration procedure. The statistical results for the DH parameter identifications for each point and each joint are shown in the Fig. 3. The results indicate that the identification process was successful, and the method ensured the calculated DH parameters converged to the correct nominal values of the DH parameters for all three points. This can be further confirmed by analyzing the statistics of the calculated DH parameters, which shows that the deviation from the nominal values is practically negligible. We have also calculated the error between end-effector positions using parameters $\widehat{P_{DH}}$ and nominal parameters $P_{DH}$ for a large random set of joint configurations spread over the robot workspace (see Fig. 4 and Table 2). The average error was calculated by computing the mean of the errors obtained from all the tested configurations. We can see that the average and maximal errors are very small. The simulation results demonstrate that the end-effector position error is negligible for all tested configurations, providing further support for the effectiveness of the proposed calibration approach.

**Table 2:** Statistics of the end-effector position error.

Set	Average position error $\bar{{\varvec{e}}}_p$ (mm)			Maximal position error $\max ({{\varvec{e}}}_p)$ (mm)		
	Mean value	Standard dev.	Maximal value	Mean value	Standard dev.	Maximal value
P1	0.00	0.00	0.02	0.01	0.01	0.03
P2	0.10	0.04	0.19	0.34	0.13	0.62
P3	0.01	0.01	0.03	0.03	0.03	0.10
P1N	0.24	0.12	0.49	0.50	0.26	1.07
P2N	0.99	0.78	3.02	2.87	2.20	8.67
P3N	0.27	0.08	0.38	0.87	0.22	1.19

For effective compensation of errors attributed to specific sources, their contribution to the position error must be significant compared to the contribution of the measurement noise. These factors can be conveniently defined and evaluated in a simulation environment. In this work, we assumed that the end-effector accuracy was similar to that of a measurement accuracy of a motion capture system. To simulate noisy data, we added a normally distributed random noise with a mean of 0 mm and a standard deviation of 0.3 mm to the position of the robot end-effector. This noise level was chosen based on the accuracy of our motion capture system^9,10^. So, the simulation was designed to reflect the noise level typically encountered in practical applications. The choice of this noise level ensured that the impact of measurement noise on calibration results was minimal and that the effectiveness of the proposed calibration approach could be accurately evaluated.

Despite incorrect DH parameters and measurement noise, the Figs. 3 and 4 clearly demonstrate that the proposed identification approach still yields satisfactory results. The average error and maximum error obtained from the proposed calibration approach are comparable to the measurements published in^10^, indicating that the proposed approach can effectively calibrate the robot’s kinematic parameters even in the presence of noise and inaccuracies. These results highlight the robustness and reliability of the proposed approach and suggest that it could be applied to a wide range of practical applications with varying degrees of complexity and noise levels. Overall, these findings provide strong support for the proposed approach’s effectiveness in accurately identifying a robot’s kinematic parameters.

The proposed calibration approach provided accurate results for most configurations, with the only notable deviation occurring in the case of P2N as seen in Fig. 4. Further analysis revealed that this deviation was likely due to insufficient data coverage in some joints. It is worth noting that the representation of configurations within each point was not uniform, primarily due to the redundancy of the robot and its ability to achieve arbitrary configurations but also due to the use of a random motion generator. This uneven distribution of data highlights the importance of selecting an appropriate set of configurations for calibration and ensuring adequate coverage of the joint space. Overall, these results highlight the importance of careful data selection and analysis when calibrating the kinematic parameters of a robot and emphasize the need for data that is as well represented as possible over the entire range of motion.

## Results of robot calibration

We performed a series of experiments using a Franka Emika Panda robot to validate the simulation results and obtain real-world measurements. We performed multiple measurement repetitions for each point (P1, P2, and P3), taking five repetitions for each point. To validate the results independently and compare them with those from^10^, we equipped the robot with a specially designed tool with markers on the end effector and a spherical joint to close the kinematic chain. Our Motion Capture (MoCap) setup incorporates 16 Prime 13 W IR cameras strategically positioned within the space, ensuring coverage exceeds the robot’s operational range. When appropriately calibrated, has a marker position measurement accuracy of better than $+/-$0.3 mm in the measurement volume^10^. The experiment setup can be seen in Fig. 2. The robot was controlled using a control system based on Matlab and ROS, which allowed for easy motion programming, kinesthetic guidance, and state acquisition. This allowed us to efficiently collect and analyze the data, ensuring that we obtained accurate results.

To ensure comprehensive coverage of the joint space, we proposed using kinesthetic guidance for data capture when performing calibration on a real robot. With this approach, the operator manually moves the robot to reach all possible and achievable configurations, allowing for a more complete representation of data across the entire range of motion. This is critical for the accurate calibration of the kinematic parameters. Additionally, this approach enables the operator to identify any unexpected issues or limitations in the robot’s motion, which can be further analyzed and potentially addressed during the calibration process.

**Figure 5:**
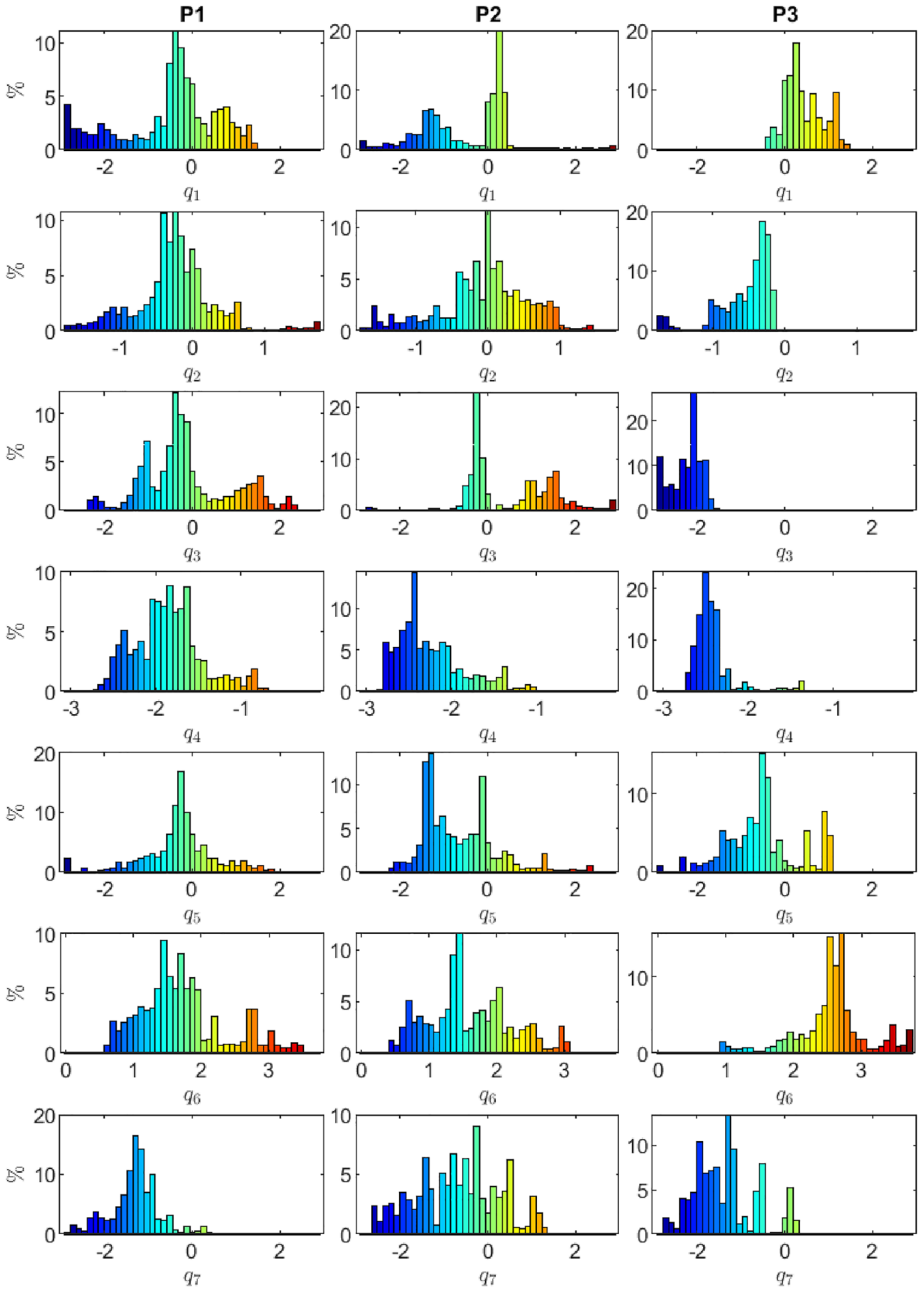
Histogram showing the percentage representation of data range for points P1, P2 and P3 for each robotic joint achieved through the use of kinesthetic guidance for data capture.

In Fig. 5, we present the percentage representation of data range for each robotic joint achieved through the use of kinesthetic guidance for data capture. As shown in Fig. 5, this approach provides a relatively uniform distribution of data across the entire range of motion for each joint, with no significant gaps or data clusters for P1 and P2. On the other hand, the data distribution for the P3 shows that for joint $q_2$ and $q_3$ data is not distributed over the whole area. This indicates that the calibration process will result in better accuracy for points P1 and P2, but less for point P3.

**Figure 6:**
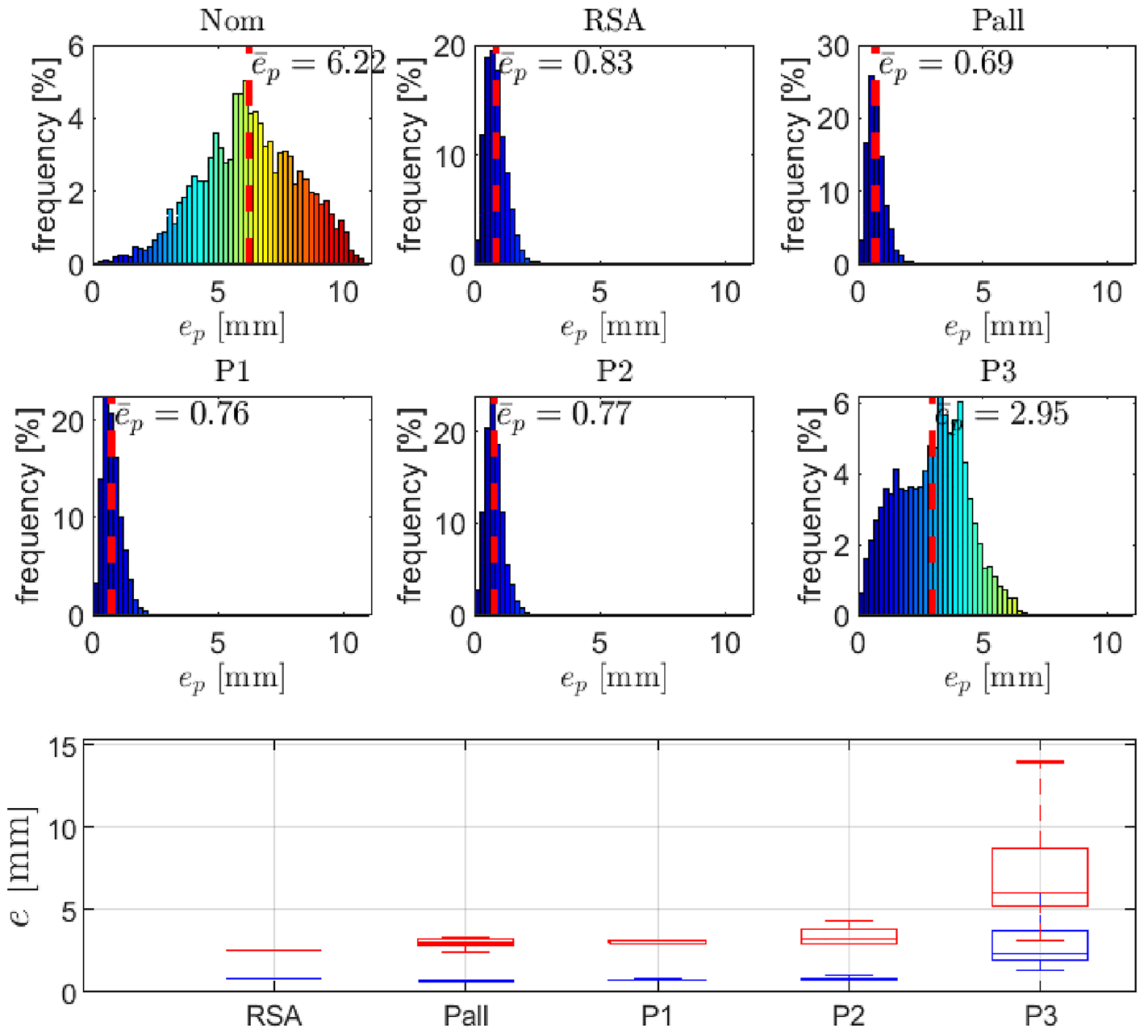
Position errors using the complete spherical joint data-set: the distribution of position errors (top and middle row), position error statistics (bottom row; blue represents mean error and red represents maximal error; note that for the RSA datataset, we have only one measurement available, whereas for the other datasets, we conducted five measurements.

DH parameters were first identified by optimization for each of the three individual points (P1, P2, and P3), and this procedure was repeated five times. Then, the measurements were combined to obtain five data sets for all three points, denoted by Pall. To compare the performance of the proposed method, we used the full data set containing all points from all five measurements. Typical results for each DH parameter identification are shown in the Fig.6. For comparison, the results with DH parameters from the^10^ are also shown in the figure. Note that the same robot was used in both experiments.

**Table 3:** Statistics of the end-effector position error using the complete spherical joint data-set. Note that for the RSA datataset, we have only one measurement available, whereas for the other datasets, we conducted five measurements.

Set	Average position error $\bar{{\varvec{e}}}_p$ (mm)			Maximal position error $\max ({{\varvec{e}}}_p)$ (mm)		
	Mean value	Standard dev.	Maximal value	Mean value	Standard dev.	Maximal value
RSA	0.83	–	–	2.57	–	–
Pall	0.70	0.01	0.72	2.98	0.34	3.35
P1	0.77	0.07	0.89	3.07	0.12	3.17
P2	0.84	0.12	1.05	3.47	0.60	4.39
P3	2.95	1.81	6.02	7.24	4.04	13.99

The obtained results in Fig. 6 and Table 3 demonstrate the effectiveness of the proposed method and confirm its success in accurately calculating DH parameters. In particular, considering all three series of measurements (P1, P2, and Pall), the results are comparable to the method used in^10^, demonstrating the reliability of our approach. However, it is worth noting that the results are slightly worse when only the P3 measurement series are used. This result was expected since the data representation for joints $q_2$ and $q_3$ in this set is limited. Despite this limitation, the proposed method has comparable performance compared to existing approaches that require more expensive hardware in terms of accuracy and reliability, highlighting its potential for various real-world applications.

**Figure 7:**
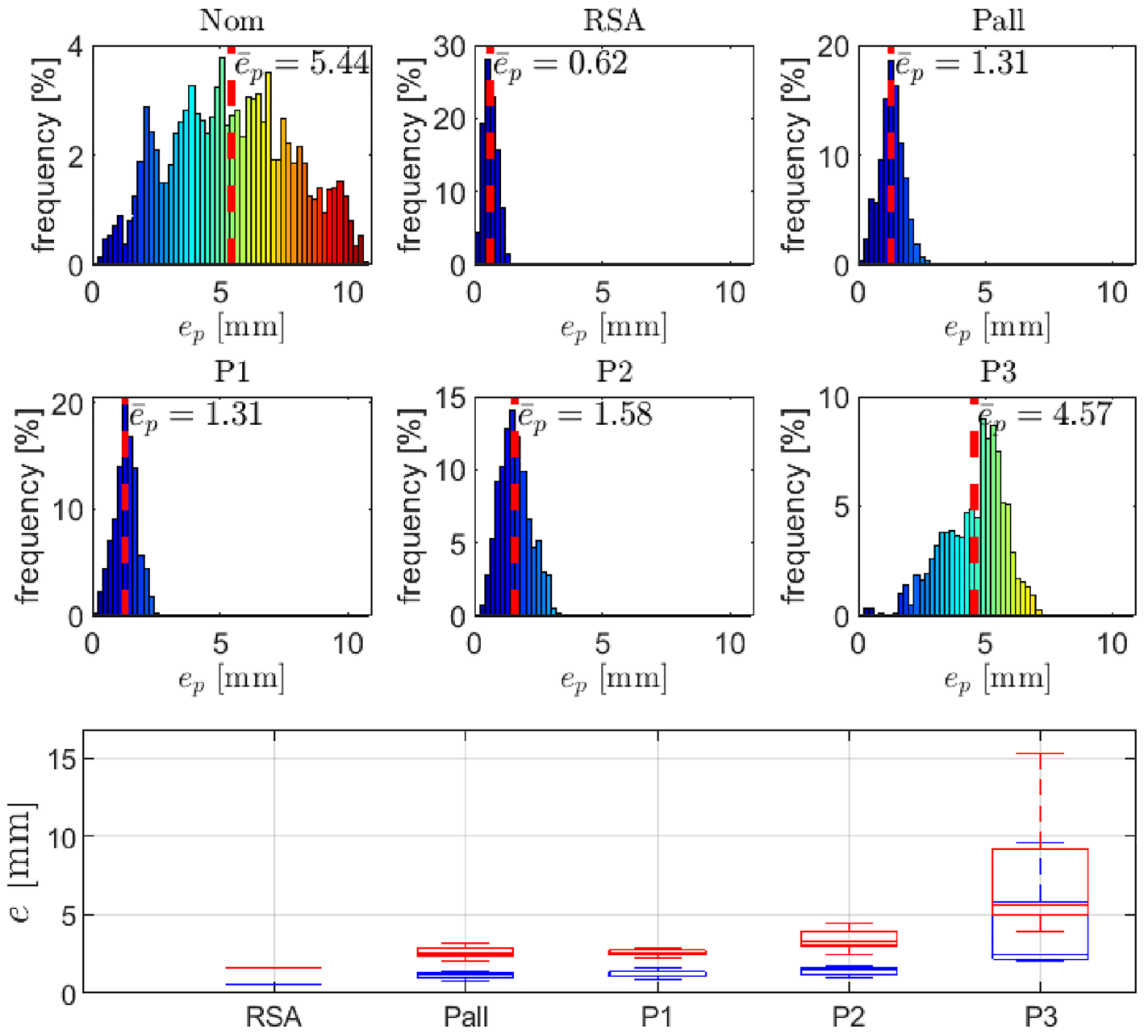
Position errors using RSA dataset reported in^10^: the distribution of position errors (top and middle row), position error statistics (bottom row; blue represents mean error and red represents maximal error; note that for the RSA datataset, we have only one measurement available, whereas for the other datasets, we conducted five measurements.

Furthermore, the results obtained from the validation of the proposed method on a RSA dataset, as reported in^10^, are presented in Fig. 7 and Table 4. As expected, the figure highlights the successful calibration of the P1, P2, and Pall datasets using our approach. It is worth noting that when compared to the results obtained from DH parameter identification in^10^, the accuracy is slightly lower. However, the remarkable performance of our method is still noteworthy, especially considering the relatively low cost of the equipment required for calibration compared to that of the equipment used in other methods.

**Table 4:** Statistics of the end-effector position error using RSA dataset reported in^10^. Note that for the RSA datataset, we have only one measurement available, whereas for the other datasets, we conducted five measurements.

Set	Average position error $\bar{{\varvec{e}}}_p$ (mm)			Maximal position error $\max ({{\varvec{e}}}_p)$ (mm)		
	Mean value	Standard dev.	Maximal value	Mean value	Standard dev.	Maximal value
RSA	0.62	–	–	1.67	–	–
Pall	1.15	0.22	1.39	2.58	0.43	3.17
P1	1.23	0.25	1.60	2.57	0.24	2.89
P2	1.44	0.28	1.71	3.45	0.71	4.43
P3	4.19	3.21	9.64	7.46	4.53	15.28

**Figure 8:**
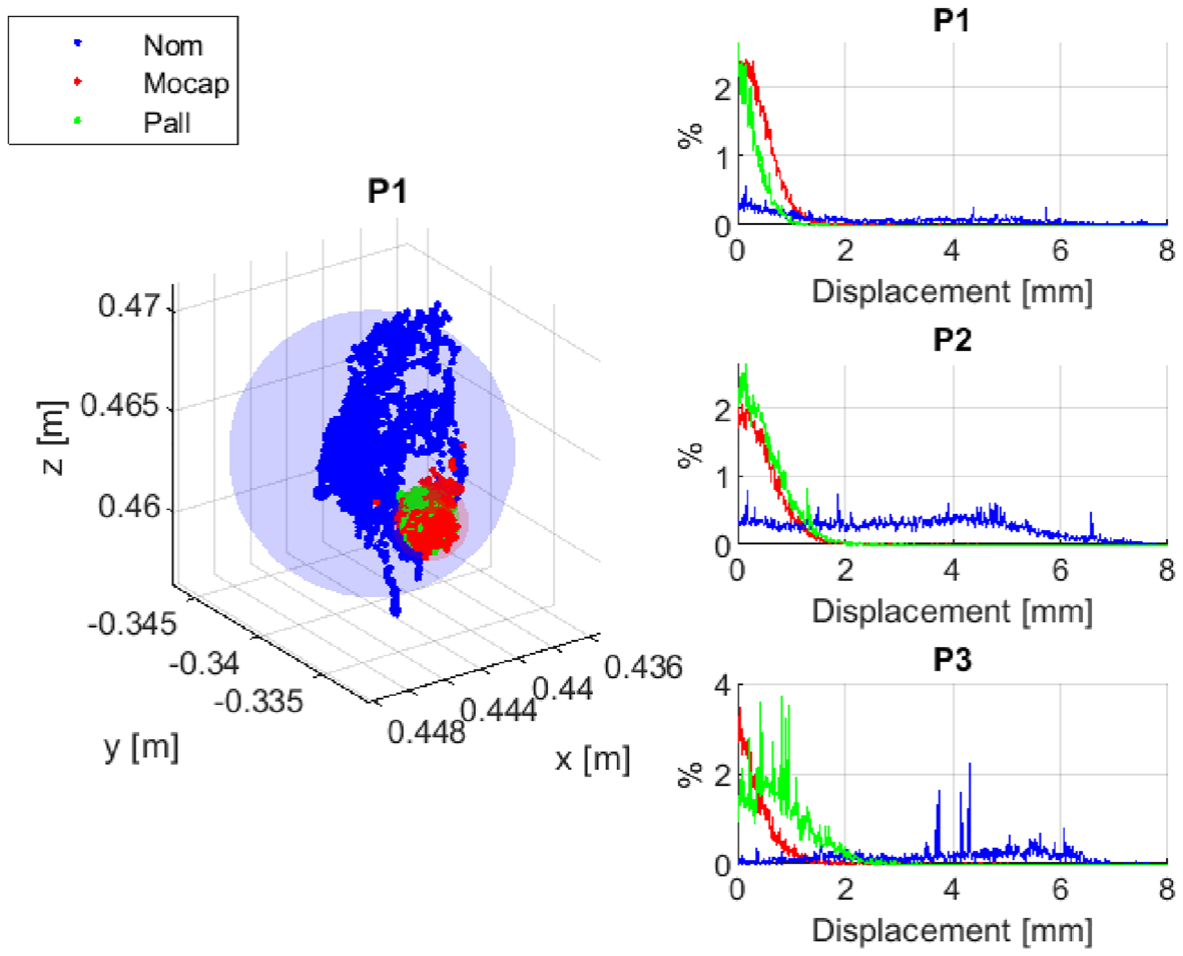
Positions of robot end-effector in point P1 measured with mocap system (Mocap), computed using nominal DH parameters (Nom) and computed using the identified DH parameters based on Pall data-set (Pall).

Figure 8 provides a visual representation of the performance of the proposed method. Specifically, the figure showcases the positions of the robot end-effector as measured by a mocap system and computed using nominal DH parameters, as well as those computed using the identified DH parameters based on the Pall dataset. As can be seen from the figure, the measured data obtained from the mocap system match closely with the calculated data based on the identified DH parameters derived from the Pall dataset, indicating the high accuracy and reliability of our proposed approach. This outcome serves as strong evidence for the effectiveness of our proposed method in accurately estimating the DH parameters for various robotic systems.

## Conclusion

In this work, we have presented a novel approach for the calibration of collaborative robots using a closed kinematic chain over a spherical joint. The proposed approach offers several notable contributions, including a numerical approach to the calibration of robotic systems and an analysis on the required joint range in the null-space needed for identification.

An evaluation of DH parameter estimation in both simulations and real robot scenarios was performed, demonstrating the high accuracy and reliability of the proposed method. The results of this study show that the proposed approach can provide a cost-effective and practical method for calibration, which has promising implications for the future development of collaborative robots.

To expand the scope of our approach, potential future work could involve adapting the proposed method to different types of joints, such as prismatic or revolute joints. Additionally, it would be valuable to optimize and validate the method on a wider range of robotic systems and scenarios, including those with suboptimal joint null-space behavior or measurement data uncertainties. Furthermore, exploring the integration of this approach with other calibration techniques could be an interesting avenue to pursue.

## Data Availability

The datasets used and/or analysed during the current study available from the corresponding author on reasonable request.
